# 204. Clinical Outcomes with Ceftaroline Monotherapy versus Daptomycin-Ceftaroline Combination Therapy in the Treatment of Methicillin-Resistant *Staphylococcus aureus* Bacteremia

**DOI:** 10.1093/ofid/ofab466.406

**Published:** 2021-12-04

**Authors:** Gabriela Andonie, Elizabeth O Hand, Kelly R Reveles, Kristi A Traugott

**Affiliations:** 1 University Health, Marrero, Louisiana; 2 University Health System, San Antonio, TX; 3 University of Texas at Austin, San Antonio, TX

## Abstract

**Background:**

Methicillin-resistant *Staphylococcus aureus* (MRSA) bacteremia is associated with poor outcomes and increased mortality. Daptomycin (DAP) and ceftaroline (CPT) in combination has been explored as a potential treatment option and showed improved outcomes compared to vancomycin/standard therapy. CPT monotherapy has been evaluated as salvage therapy for MRSA bacteremia but, to our knowledge, not as a comparator to DAP-CPT combination therapy. The purpose of this study is to compare the clinical outcomes of DAP and CPT combination therapy to CPT monotherapy in the setting of MRSA bacteremia.

**Methods:**

A retrospective chart review of adult patients (≥ 18 years of age) admitted to University Health from January 2017 to December 2020 with a diagnosis of MRSA bacteremia was performed. Patients received either CPT monotherapy or DAP-CPT combination therapy for a minimum of 48 hours during their course of therapy.

**Results:**

Thirty-two patients met inclusion criteria and were evaluated. Primary source of infection was pulmonary in the CPT monotherapy group (n=7/24; 29.2%) and osteomyelitis in the DAP-CPT combination group (n= 4/8; 50.0%). Median duration of bacteremia was 8 days and 9 days in the CPT monotherapy and DAP-CPT combination group, respectively. Microbiological cure was achieved in 95.8% (n=23/24) of patients in the CPT monotherapy and 100% (n=8/8) of patients in the DAP-CPT combination group. Bacteremia relapse (30 day, p=0.62; 60 day, p=0.63), readmission rates (30 day, p=0.62; 60 day, p=0.63), and mortality rates (30 day, p=0.70; 90 day, p=0.85) were similar in both groups. There was no statistically significant difference in safety parameters, including incidence of acute kidney injury (p=1.00) and creatine kinase elevations (p=1.00). Bone marrow suppression after at least 72 hours of therapy, including anemia, leukopenia, and thrombocytopenia, was also not statistically significant between groups.

**Conclusion:**

This study was unable to find a statistically significant difference in clinical outcomes between patients receiving CPT monotherapy or DAP-CPT combination therapy. A large prospective, randomized controlled trial to assess CPT monotherapy and DAP-CPT combination therapy for the treatment of persistent MRSA bacteremia is warranted.

**Disclosures:**

**All Authors**: No reported disclosures

